# A double-blind clinical trial to compare the efficacy and safety of a multiple amino acid-based ORS with the standard WHO-ORS in the management of non-cholera acute watery diarrhea in infants and young children: “VS002A” trial protocol

**DOI:** 10.1186/s13063-022-06601-5

**Published:** 2022-08-25

**Authors:** Rina Das, Rukaeya Amin Sobi, Al-Afroza Sultana, Baitun Nahar, Pradip Kumar Bardhan, Laura Luke, Olivier Fontaine, Tahmeed Ahmed

**Affiliations:** 1 International Centre for Diarrheal Disease Research, Bangladesh (icddr,b), Dhaka, Bangladesh; 2 Department of Environmental Health Sciences, Rollins School of Public Health, Emory University, Atlanta, GA 30322 USA; 3 Science & Technology, Entrinsic Bioscience Inc., Boston, MA USA; 4 James P. Grant School of Public Health, BRAC University, Dhaka, 1212 Bangladesh; 5 Department of Global Health, University of Washington, Seattle, WA 98104 USA

**Keywords:** VS002A, WHO-ORS, Diarrhea, Randomized, Amino acid-based ORS

## Abstract

**Background:**

Diarrhea is the second deadliest disease for under-five children globally and the situation is more serious in developing countries. Oral rehydration solution (ORS) is being used as a standard treatment for acute watery diarrhea for a long time. Our objective is to compare the efficacy of amino acid-based ORS “VS002A” compared to standard glucose-based WHO-ORS in infants and young children suffering from acute non-cholera watery diarrhea.

**Methods:**

It is a randomized, double-blind, two-cell clinical trial at Dhaka Hospital of icddr,b. A total of 312 male children aged 6–36 months old with acute non-bloody watery diarrhea are included in this study. Intervention arm participants get amino acid-based ORS (VS002A) and the control arm gets standard glucose-based WHO-ORS. The primary efficacy endpoint is the duration of diarrhea in the hospital.

**Discussion:**

Oral rehydration therapy (ORT) with the present ORS formulation has certain limitations - it does not reduce the volume, frequency, or duration of diarrhea. Additionally, the failure of present standard ORS to significantly reduce stool output likely contributes to the relatively limited use of ORS by mothers as they do not feel that ORS is helping their child recover from the episode of diarrhea. Certain neutral amino acids (e.g., glycine, L-alanine, L-glutamine) can enhance the absorption of sodium ions and water from the gut. By using this concept, a shelf-stable, sugar-free amino acid-based hydration medicinal food named ‘VS002A’ that effectively rehydrates, and improves the barrier function of the bowel following infections targeting the gastrointestinal tract has been developed. If the trial shows significant benefits of VS002A use, this may provide evidence to support consideration of the use of VS002A in the present WHO diarrhea management guidelines. Conversely, if there is no evidence of benefit, these results will reaffirm the current guidelines.

**Trial registration:**

ClinicalTrials.gov NCT04677296. Registered on December 21, 2020.

**Supplementary Information:**

The online version contains supplementary material available at 10.1186/s13063-022-06601-5.

## Background

Diarrheal disease is the second deadliest disease for under-five children globally and the situation is more serious in developing countries. Oral rehydration solution (ORS) is being used as a standard treatment for acute watery diarrhea for a long time [1]. The ORS currently recommended by the WHO/UNICEF contains glucose, sodium chloride, potassium chloride, and tri-sodium citrate dehydrate, which is optimal for rehydration of patients of all ages with dehydration from acute diarrhea of any etiology [2]. However, the present ORS formulation has certain limitations [3]. It does not reduce the volume, frequency, or duration of diarrhea. Additionally, the failure of present standard ORS to reduce dramatically stool output likely contributes to the relatively limited use of ORS by mothers as they do not feel that ORS is helping their child from the episode of diarrhea [4]. Thus, it warrants the development of a newer and improved formulation of ORS to become more effective against diarrhea.

Adding neutral amino acid or their peptides can enhance the absorption of sodium ions and water, an approach to developing an improved ORS formulation by adding neutral amino acid or their peptides to WHO-ORS [5]. Enterade® is a proprietary blend of five amino acids (threonine, valine, serine, tyrosine, and tryptophan) that acutely restores water and electrolyte losses by facilitating intestinal sodium and water transport with similar stoichiometry to glucose [6], but without stimulating chloride secretion like glucose [7]. Based on previous findings regarding amino acid-based ORS, a sugar-free self-stable amino acid-based hydration medicinal food named VS002 was developed, which effectively rehydrates and improves the barrier function of the bowel following infections targeting the GI tract [8]. By using this concept, the University of Florida (UF), developed a sugar-free, shelf-stable amino acid-based hydration medicinal food named “VS002A” that effectively rehydrates, and improves barrier function of the bowel following infections targeting the gastrointestinal tract. So, we aimed to know whether VS002A will be superior or not to WHO-ORS in the treatment of acute non-cholera watery diarrhea in infants and young children.

### Gut health biomarkers

Diarrheal disease among under 5 children contributes to 43% of stunted growth and impaired cognitive development, affecting one-fifth of children worldwide and one-third of children in low- and middle-income countries (LMICs) [9, 10]. A vicious cycle of enteric infection and malnutrition often leads to enteropathy for extended periods in young children [9, 11]. This type of enteropathy is known as environmental enteric dysfunction (EED). EED is a sub-acute inflammatory condition of the small intestinal mucosa of unknown etiology [12]. It is characterized by structural changes in the small intestine including villous atrophy and crypt hyperplasia compromising nutrient absorption and pathogenic barrier (increased permeability and inflammatory cell), impaired gut immune function, malabsorption, growth faltering, and generally asymptomatic, as distinct from the diarrheal disease [11, 13]. Citrulline (CIT) is an amino acid produced by intestinal epithelial cells and found lower in patients with enteropathy [14]. Tryptophan (TRP), a plant-derived essential amino acid (EAA) is needed to support growth and health in humans. In response to infection, TRP is mostly catabolized by an enzyme indoleamine 2,3-dioxygenase (IDO) to toxic metabolite kynurenine (KYN). Low plasma TRP, high KYN, and elevated tryptophan-kyanurenine (KT) ratio are found to be associated with infections and chronic immune activation [10].

Data are limited from LMICs including Bangladesh on inflammatory and pathological changes in the gut wall like EED among children with diarrhea. In this study, we also evaluate plasma CIT concentration as a potential marker of functional enterocyte mass and absorptive functions of the intestine, and KT ratio as a marker of gut inflammation.

## Methods

### Aim

The main aim of the VS002A trial is to compare the efficacy (duration of diarrhea, stool output, ORS intake, and clinical success) of amino acid-based ORS “VS002A” compared to standard glucose-based WHO-ORS (GORS) in infants and young children suffering from acute non-cholera watery diarrhea in a superiority trial. We specify our objective as (i) to evaluate and compare clinical responses (duration of diarrhea [primary end point], stool output, ORS intake, and clinical success [exploratory endpoints]) in infants and young children suffering from acute non-cholera watery diarrhea treated with standard glucose-based WHO-ORS and amino acid-based ORS “VS002A” in a superiority trial and (ii) to evaluate and compare exploratory endpoints of electrolyte imbalance as possible complications in infants and young children suffering from acute non-cholera watery diarrhea treated with standard glucose-based WHO ORS and amino acid-based ORS “VS002A.”

We have our secondary objective to investigate the plasma concentration of CIT and KT ratio among the children having dehydrating diarrhea and examine associations between concentrations of CIT and KT ratio with concurrent factors.

### Study design

The VS002A trial is a randomized, double-blind, two-cell superiority clinical trial comparing WHO-ORS and VS002A conducted in 312 male children aged 6–36 months, presenting with non-bloody acute non-cholera diarrhea with some dehydration in the Dhaka Hospital of icddr,b.

**Fig. 1:**
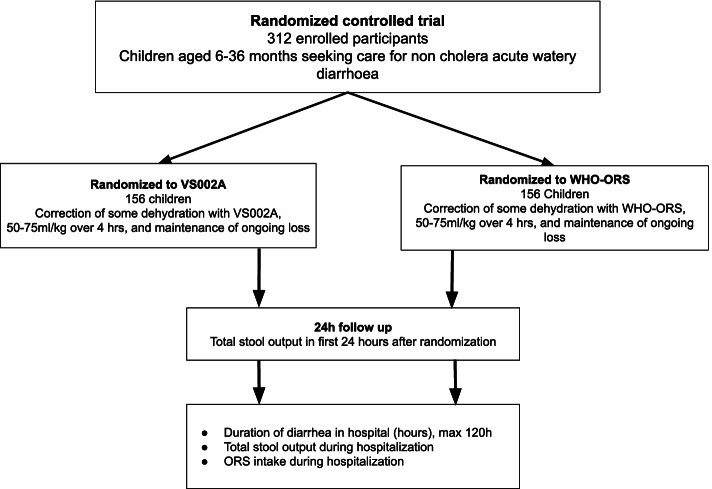
VS002A trial study design

The trial protocol was developed by the investigators of icddr,b, Nutrition and Clinical Services Division, Dhaka Bangladesh together with Entrinsic Bioscience. The study design is described in Fig 1.

### Study setting and population

The study is implemented in Dhaka Hospital of icddr,b. The study subjects are 312 male children (to facilitate separate collection of urine and stool), admitted to Dhaka Hospital of icddr,b.

### Inclusion criteria

1. Age: 6–36 months
2. Duration of diarrhea ≤48 h
3. Some dehydration (judged clinically according to the “Dhaka method”) [15]
4. Written informed consent by either parent/guardian

### Exclusion criteria

1. Severe malnutrition (weight-for-length WLZ/weight-for-height WHZ/weight-for-age WAZ <−3 or presence of nutritional edema)
2. Patients with diarrhea due to cholera
3. Systemic illness (e.g., pneumonia, tuberculosis, enteric fever, and meningitis)
4. Bloody diarrhea
5. Any congenital anomaly or disorder (e.g., diagnosed inborn error of metabolism, congenital cardiac disease, seizure disorders, hypothyroidism, and Down’s syndrome)
6. Requirement of additional intravenous fluids after being provided with an IV for 4 h on admission if severely dehydrated
7. Has documentation of taking antibiotics and/or antidiarrheal within the last 48 h before hospitalization.

### Implementation plan

#### Screening and enrollment procedures

All male children aged 6–36 months, presenting with diarrhea (onset ≤48 h) and some dehydration are screened by the study nurses in the Dhaka Hospital of icddr,b (triage/OPD/short stay ward) for possible inclusion in the study. After getting verbal consent from the parents, all screened children are kept in the Clinical Research Ward for conducting the following activities:

- A thorough clinical history and physical examination are done, and body weight and height are measured.
- Correction of some dehydration: The child receives standard WHO-ORS for correction of dehydration, 50–75 ml/kg over 4 h, according to the rehydration protocol followed at icddr,b [2]. During this period, cholera cases are detected using a rapid screening test “Cholkit” [16], conducted by a Study Nurse.

#### Consent

After confirming eligibility, the accompanying primary caregiver is provided written informed consent for the Cholkit test. When the Cholkit test result is negative, the caregiver is provided with another written informed consent for the main trial. For illiterate caregivers, documented witnessed verbal consent and a thumbprint are obtained. During consent, the purpose of the study, as well as all the study procedures, and possible risks are explained to the caregiver by a member of the trial team in the local language. Consent is taken for the collection and storage of and use of samples collected. Caregivers are also informed that they can choose to participate in the trial but not provide samples or they can withdraw their participation at any time after enrollment in the study.

#### Sequence generation

Upon meeting eligibility criteria for the study and after obtaining written informed consent, the subjects are individually randomized into one of the two study groups, VS002A vs. WHO-ORS, through a variable permuted block procedure. Enrolled patients then receive an identification number according to their order of admission in the study. Subjects are then randomized into one of the four groups, using a predefined allocation table. Enrolled patients receive the serially numbered test product that corresponds to their identification number.

#### Allocation concealment

Envelopes containing the treatment allocation are opened by the study physician on participant enrolment. To be robust, the envelopes will be truly opaque, sequentially numbered, and opened in the correct order. Treatment allocation (once assigned) will remain blinded to the participant, the site Principal Investigator, co-Investigators, the site study staff, and the study physicians during all data collection phases of the study.

#### Blinding (masking)

Entrinsic Bioscience blinds the study product. The study products (VS002A and WHO-ORS) are packaged in 310 mL Tetra Pak cartons made indistinguishable except for randomized manufacturer serial numbers broken out into 2 groups, with 2 separate lot numbers per treatment group identifiable by their serial number prefix (e.g., D-XXXX; G-XXXX; O-XXXX; U-XXXX). The entire product group consists of either VS002A or the WHO-ORS. A master file linking groups to product identity is generated; one kept by a single individual at Entrinsic Bioscience and one kept by the Senior Pharmacist of icddr,b pharmacy. The identity of the specific product is blinded to the subjects, the icddr,b Dhaka Hospital staff, sponsor, and the investigators. Patients are truly blinded to the interventions due to similar color, smell, taste, and flavor (citrus) between VS002A and WHO-ORS. The final unblinding of product categories will occur only after statistical analysis.

#### Enrollment and interventions

After signing an informed consent document, participants are included in the study and are allocated into one of the 2 ORS groups (156 children in each group) according to a pre-determined randomization schedule at icddr,b. The enrolled children are then randomized into four groups: two VS002A vs. two WHO-ORS. Both groups are receiving standard care for diarrheal disease, including rehydration, supplemental zinc, nutritional counseling, follow-up, and guidance on when to return, as per the WHO guidelines. We expect to enroll approximately 20 patients per month in Dhaka Hospital to reach a target enrollment of 312 children within the study period.

i) Composition of the 2 different oral rehydration solutions (Table 1)

ii) Safety data of advanced amino acid-based ORS: VS002A

**Table 1:** The composition of the two ORSs to be tested are (i) WHO-ORS: standard low-osmolarity glucose-based ORS (as control) and (ii) VS002A: advanced amino acid-based ORS

Compositions	WHO-ORS	VS002A
Sodium (mmol)	75	67.1
Potassium (mmol)	20	20.1
Chloride (mmol)	65	49.4
Citrate (mmol)	10	14.2
Calcium (mmol)	-	1.2
Magnesium (mmol)	-	1.2
Amino acids (mmol) composition	-	57.2
		Aspartic acid, glycine, serine, threonine, tyrosine, alanine, arginine, proline
Glucose (mmol)	75	-
Osmolarity (mOsmol)	245	212

Entrinsic Biosciences amino acid formulas are fully compliant with FDA 21 CFR requirements. The details of the safety data of VS002A are attached as the supplementary file.

#### Treatment procedures

The study patients are placed on diarrhea cots. Initial treatment dosing with WHO-ORS or VS002A is estimated using bodyweight following 5–10 ml/kg after each loose stool as per icddr,b guideline, to maintain the ongoing loss. Adjustments may be required and hydration is maintained using smaller or larger volumes depending on measured losses. Children are fed using a spoon. Assessment and treatment are to continue until the resolution of diarrhea. The child is supplemented with tablet zinc: one tablet (20 mg) per day for 10–14 days [2].

In young children who cannot control micturation, a pediatric urine collection (PUC) bag is applied to facilitate the separate collection of stool and urine.

The patient receives liquid, semi-solid, and solid foods appropriate for age. Breast-fed children continue to receive breast milk. If stool culture reveals any pathogen, then the patients receive an appropriate antibiotic if applicable and are excluded from the study (e.g., azithromycin in cholera). Intakes of ORS, food, and medicines are supervised by a nurse and are recorded. Vital signs are recorded for each patient every 4 h. Intake of ORS and water is recorded every 4 h while outputs of stool and urine and vomitus, and stool frequency are recorded continuously. Clinical assessment of the study children is performed by the study investigators and/or designated research physicians. Vital signs and body weight are measured and recorded by experienced nurses of the Study Ward. And other measurements, e.g., stool frequency and volume/weight, intake of rehydration fluids, and urine outputs are measured by designated health workers under the supervision of the study nurse who would record them. Each participating child is closely monitored for the occurrence of any adverse events such as worsening diarrhea or increased vomiting, and their severity and duration are noted and information is also recorded for analysis.

#### Standard management

All two groups will receive standard of care for diarrheal disease, including zinc, rehydration, and nutritional counseling following WHO guidelines. Children with some dehydration will be rehydrated and stabilized. As part of standard case management, caregivers will be advised to seek care immediately if their child is unable to drink or breastfeed, develops a fever, starts passing blood in the stools with continued diarrhea, or becomes sicker.

#### Post-trial care

In addition, as per standard WHO guidelines, the caregiver will also be advised to bring their child back to the health facility if the child’s clinical status is deteriorating after discharge from the hospital for further treatment.

### Dissemination policy

We will be planning to disseminate the result of the trial findings to international and national scientific members and policymakers of the Bangladesh Government after the completion of the trial. And we will plan to publish our study findings in international peer-reviewed journals.

### Outcomes

#### Primary outcomes

Duration of diarrhea in hospital (hours)

#### Exploratory outcomes

- Stool output in the 1st 24 h of hospitalization (g/kg body wt.), further divided into two 12-h periods
- Total stool output during hospitalization (g/kg body wt.)
- ORS intake in the 1st 24 h of hospitalization (g/kg body wt.)
- Total ORS intake (ml/kg body wt.)
- Unscheduled IV (frequency/ORS group)
- Treatment failure (frequency/ORS group)
- Output and frequency of vomiting
- Change in body weight (between pre-randomization and post-treatment)
- Urine output in the 1st 24 h of hospitalization (g/kg body wt.)
- Total urine output during hospitalization (g/kg body wt.)
- Documented infectious agent
- Blood chemistry (between pre-randomization and after 24 h of treatment)
- Gut health biomarker: citrulline (CIT) and kynurenine to tryptophan ratio (KT) between pre-randomization and at 24 hours after the enrolment)

### Trial assessments

Children enrolled in the trial are followed up for up to 120 h post-enrolment or until the first primary endpoint is reached which means diarrhea resolution, whichever is earlier. The schedule of enrolment, intervention, and outcome assessments is shown in Table 2, which follows the SPIRIT (statement provides evidence-based recommendations for the minimum content of a clinical trial protocol) guidelines Table 4.

**Table 2:**
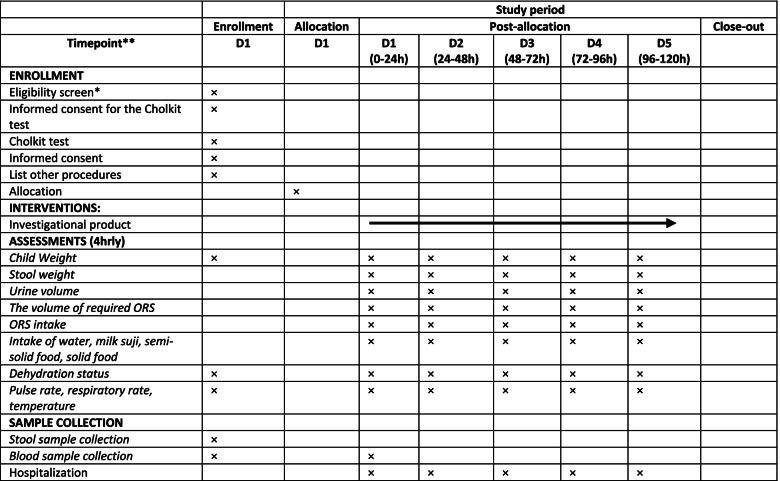
Schedule of enrollment, intervention, and outcome assessments for VS002A trial

*Screening is expected to be completed on the same single day; stool samples are only collected on D1 and blood samples are collected on D1 and D2 (blood samples for gut health biomarkers are collected for 100 patients); *D=* day; Cholkit test: rapid test for detection of *V. Cholerae*

### Laboratory tests

#### Routine

Blood is obtained by venipuncture to determine serum electrolytes, and blood glucose is determined on admission and 24 h after enrolment. Complete blood count, serum creatinine, stool routine examination, stool bacterial culture, and Rota viral antigen for all patients are done on admission only. Other clinical laboratory investigations, e.g., serum creatinine, blood culture, urine microscopy/culture, stool electrolytes, and chest X-ray, may be performed and repeated if clinically indicated.

#### Gut health markers

In a sub-sample of the first 100 children, plasma vitrulline (CIT) and kynurenine-tryptophan (KT) ratio are measured to have an idea about the changes associated with environmental enteric dysfunction (EED). The tests are done on enrolment and after 24h of the enrollment at icddr,b lab, using the serum collected for routine blood testing, therefore no extra amount of blood or venipuncture is required. Quantitative analyses of plasma CIT concentrations are performed at icddr,b using the enzyme-linked immunosorbent assay (ELISA) method. KT is also be tested at icddr,b using liquid chromatography/quadrupole time-of-flight mass spectrometry (Q-TOF LC-MS) platforms [17].

### Discontinuation procedure

#### Criteria for removal from study and treatment failure

- Patients are discharged from the hospital when diarrhea is resolved (the working definition of resolution of diarrhea has been mentioned below). However, no participants are discharged before 24 h from enrolment.
- If it takes more than 5 days (≥ 120 h) to resolve diarrhea or if a patient becomes dehydrated requiring unscheduled IV fluids, we transfer the patient to the general in- patient’s ward for further evaluation and treatment. In that case, it is considered a “treatment failure.”
- After enrollment, if any patient develops any co-morbidity like pneumonia, sepsis, electrolyte imbalance or requires specific antibiotics and/or special attention/intervention, we transfer the patients to the General ward or ICU of the hospital and are removed from the study. The reason for removal is recorded and an off-study document is completed by the study team.
- Patients who decide to withdraw from the study are defined as “drop-out.”
- Patients who do not consume at least 80% of ORS offered are considered “non-compliant”.
- If stool culture reveals an agent that needs antibiotic therapy, the patient is removed from the study, and an off-study document is completed by the study team.
- All data collected on randomized subjects are kept for intent-to-treat analysis, including patients who withdrew from the study (drop out), have co-morbidity, or are defined as treatment failures or non-compliant.

#### Definitions

- Duration of diarrhea in hospital: time in hours, from randomization till resolution of diarrhea.
- Resolution of diarrhea: the passage of the last liquid or semi-liquid stool before one soft/formed stool or no stool for 12 h [18].
- Stool output: The weight of stool in g/kg of admission body weight expressed per time period (i.e., per 24 h and for the entire duration of diarrhea).
- ORS and plain water intakes: The total volume (in ml) of ORS or plain water is taken per kg of admission body weight, expressed per time period.
- Treatment failure: If diarrhea continues for more than 120 h after enrollment, or requires unscheduled IV fluids due to progressive dehydration, requiring transfer to the general in-patient ward
- Un-scheduled IV: When intravenous rehydration fluids are given due to the inability of the ORS to maintain hydration
- Non-compliance: inability to consume ≥ 80% of ORS

#### Recording of adverse events

All adverse events regardless of the seriousness, severity, or relationship to the study medication are recorded on the adverse events pages of case record forms. Adverse events that meet the definition of a serious adverse event are to be reported on the serious adverse event form provided for this study. Adverse events will be documented in clear, unambiguous medical language. All adverse events not resolved by the end of the study or that have not been resolved upon the subject’s discontinuation in the study will be followed until the event resolves, the event stabilizes, or the event returns to baseline if a baseline value is available. All serious adverse events are to be reported to the secretariat of the Ethics Review Committee of icddr,b within 24h of the investigator or their staff becoming aware of them. Reporting is performed by recording as much information as is available at the time on the serious adverse event (SAE) form by icddr,b IRB.

### Quality assurance

The following measures provide quality assurance:

- An extensive initial and subsequent ongoing training sessions for study staff in the protocol procedures with a focus on dehydration assessment to ensure the reliability between study staff is high.
- Real-time electronic data capture in the ClaimIt portal ensures data validation, such as range, logical checks, and data integrity.
- Good clinical practice (GCP) training and certification are compulsory for all study staff.
- Study Investigators receive brief weekly progress reports from the data management assistant during the entire study period and participate in regular weekly zoom meetings with donor and CRO. The weekly progress reports include the number of children screened, number of children enrolled, number of treatment failures, number of withdrawn, number of drops out, number of children who completed the study, and number of children currently admitted in the study ward conducted.
- Study Investigators are responsible for assuring that the training of the study staff is rigorous and of high quality. They schedule the testing and retraining as required. Assessment of individual study personnel’s abilities to use the standardized enrolment criteria and dehydration assessment consistently across the study population are key responsibilities of the study investigators.
- Entrinsic Bioscience, Obvio, and others identified by them will ensure structured monitoring visits conducted to the study site. The monitoring visits will have as their primary aim quality control and the improvement of study implementation. The monitors will make direct observations of all relevant study procedures and data management activities.
- The data management assistant runs a weekly set of range and consistency checks, resolves inconsistencies or queries with the sites, and provides data summaries as the trial progresses. The queries are resolved before the next set of weekly check reports.

### Compensation

No financial compensation is provided for participation in the trial.

### Data management

An external data management system (ClaimIt) ensures the harmonization of data collection and data management processes. The study site collects information on a core set of variables with standard definitions. A set of range and consistency checks are applied to these available variables. The study site is responsible for data entry and initial cleaning of the data, including running range and consistency checks, as well as periodic reviews of distributions and identification of outliers. icddr,b resolves any inconsistencies within its database, in consultation with the data collection team, and with verification if needed. The study site is required to provide data on the core set of variables in the ClaimIt database.

### Statistical considerations

#### Sample size and power

The total sample size is 312 male children. In previous non-cholera studies of children comparing an anti-diarrheal to placebo [19–22], the effect sizes for the hourly duration of diarrhea and 24-h stool volume ranged from 0.3 to 1.0 (duration) and 0.3 to 0.7 (stool output), respectively. Outcome improvements ranged from 15 to 45% with treatment (vs. placebo). A clinically significant improvement of 20% produces effect sizes of 0.4 to 0.7 (duration) and 0.3 (stool output) using the pooled standard deviations reported within each outcome. When applied to conventional alpha (0.05) and beta (0.20) values, between 32 and 174 patients per group are required for statistical significance. The maximum anticipated accrual rate of 3 patients per week over 24 months results in 156 patients per group, or 132 patients per group assuming 15% attrition. Therefore, the final anticipated patient group numbers are estimated to provide >99% and 68% power to detect a 20% difference in hourly diarrhea duration and 24-h stool output, respectively.

Justification for the content of the excerpt, based on an independent samples *t*-test (in the Data Analysis part), is reviewed in Table 3. Our sample size estimates were based on detecting a difference in the primary outcome variable (duration of diarrhea). We acknowledge that we were underpowered for secondary outcomes such as stool volume. The secondary outcomes in our study were better classified as exploratory outcomes, which is why we are not adjusting our alpha for multiple endpoints.

**Table 3:** Duration of diarrhea (days^a,b^)

Study	M_t_	SD_t_	M_p_	SD_p_	Mdiff	%diff	SDpooled	ES	20%_diff_	ES	nreq	nact	%op
Cojocaru^a^	4.05	1.48	5.73	1.77	1.68	29.3	1.62	1.0	1.14	.71	32	132	99
Santos^b^	4	2.1	4.7	2.2	0.7	14.9	2.15	0.3	0.94	0.44	81	132	99

*Mt* mean of treatment, *SDt* standard deviation of treatment, *Mp* mean of placebo, *SDp* standard deviation of placebo, *Mdiff* mean difference between treatment and placebo, *%diff* percentage of mean difference between treatment and placebo, *SDpooled* pooled standard deviation, *ES* effect size (Mdiff/SDpooled), *20%diff* units for 20% difference from placebo, *ES* effect size (20% units difference/SDpooled), *nreq* sample size required (alpha 0.05, beta 0.20), *nact* actual sample size planned for PR-17028, *%op* percentage observed power (relative to desired minimum of 80%); note – primary study outcome exceeds power criterion

^a^Cojocaru et al. (2002)

^b^Santos et al. (2000)

#### Statistical analysis

Data are collected on paper and entered electronically capturing information such as demographics, medical history, clinical assessments, clinical course, etc., and transcribed into an electronic database using Statistical Package for Social Sciences (SPSS) (version 20), STATA, or equivalent. The primary analysis will be intended to treat analysis which will include all of the patients who were randomized in the study. A per-protocol analysis will also be done only including only those randomized subjects with efficacy data on diarrhea volume and who have taken at least 80% of the investigational product. If the distribution of the variable does not have a normal distribution, a log transformation of data will be performed before the t-test analysis or a non-parametric equivalent test will be performed. Differences between the two treatment groups (VS002A versus WHO ORS) will be examined for baseline characteristics before performing a statistical analysis of the efficacy endpoints. If any differences are considered to be clinically important, subgroup analysis will be presented for the relevant endpoints. Depending upon the normality of distribution of the quantitative variables, a *t*-test or Mann-Whitney *U* test will be used to compare the duration of diarrhea, stool output, the output of vomit, and ORS intake. The chi-squared test will be used to compare treatment failures, unscheduled IV, and frequency of vomiting. Comparison of the primary outcome will be performed with and without adjustment of design effects (age stratum) and patient characteristics (cause, clinical characteristics on admission, nutritional status). All analyses will be performed separately on children as per protocol and on all children on an intention-to-treat basis. In addition, a life-table analysis will also be performed. Time to cessation of diarrhea will be compared by log-rank test, and adjustments of covariates will be made via Cox proportional hazards regression.

An interim data analysis will be performed by a third-party biostatistician once 50% of the enrollment has been completed. The interim analysis will not unblind or regroup the study population; the assessment will be done in the whole patient cohort. The analysis will include safety and adverse events, tolerability and compliance to the ORS intake, and the severity of diarrhea/duration of diarrhea after randomization (to assess the severity of the disease and determine if sample size assumptions are correct). No efficacy calculations will be performed and no comparison between groups will be done.

### Trial governance

The VS002A trial is overseen by the site Investigators and Entrinsic Biosciences and Obvio trial coordinators. This team is responsible for the overall supervision of the trial. All Serious Adverse Events are to be reported to the secretariat of the Ethics Review Committee (ERC) of icddr,b within 24h of the investigator or their staff becoming aware of them. Reporting should be performed by recording as much information as is available at the time of the Serious Adverse Event (SAE). The ERC of icddr,b will determine how the trial is overseen—they may form a Data and Safety Monitoring Plan (DSMP) to ensure the data and participants' safety regarding medical and ethical grounds.

The DSMP includes five members with expertise in clinical trials, statistics, child mortality assessment, ethics, and pediatric care in resource-limited settings. When approximately half of the participants are accrued in the study, the DSMP will review an interim data analysis by arm to determine whether stopping boundaries have been crossed. The SPIRIT checklist for the present study is provided in the additional file.

### Auditing plan

The VS002A trial will be monitored by WAGAS (Data Management, Quality Control, Additional Services), a renowned international monitoring organization. As the COVID-19 pandemic is continuing, WAGAS will be responsible for remote monitoring of the trial activities. Our designated study staff (Data scanning assistant) will scan the study-related documents along with the CRF, and consent forms and will share those via an online portal where patients' confidentiality will be maintained accordingly for remote monitoring. The study team will also provide live video of the study activities after taking proper consent from the parents of the study patients for remote monitoring.

### Protocol amendments

According to the icddr,b IRB guidelines, if there will be any protocol amendment, each and every version of the protocol along with the CRF and consent forms will be submitted to the icddr,b IRB for their approval.

## Discussion

VS002A trial is a randomized double-blind superiority trial testing the potential role of amino acid-based ORS: VS002A to the standard WHO-ORS in reducing the duration of non-cholera, and acute watery diarrhea in infants and young children.

It has been reported that the glucose contained in standard ORS may cause failure to absorb fluid and electrolytes adequately from the gut and thus worsen diarrhea in different pathophysiological ways [23, 24]. Although ORS substantially enhances glucose-stimulated sodium absorption and correction of dehydration, it does not significantly decrease stool output, which is one of the main reasons for the underutilization of ORS [25, 26]. Whereas, certain neutral amino acids (e.g., glycine, L-alanine, L-glutamine) can enhance the absorption of sodium ions and water from the gut [5, 27].

Three clinical trials comparing glutamine-containing ORS with the WHO-ORS showed that it does not have any clinical advantage in children with non-cholera diarrhea [28]. One common thing among all the trials is that all the experimental amino acid-containing ORSs also contained glucose in variable amounts (9 g/L to 20 g/L); also, most of these solutions had relatively high osmolarity (320–400 mosmol/L). One study which compared glucose-free glutamine-containing ORS with the WHO-ORS showed similar efficacy in children with acute non-cholera diarrhea [29]; it is to be noted that glutamine behaves like glucose in stimulating anion secretion [30]. A study comparing L-isoleucine supplemented glucose-containing ORS with WHO-ORS in young children with acute diarrhea, but the differences were not statistically significant [31]. However, in adult patients suffering from severe cholera, L-histidine-supplemented ORS was found superior to a histidine-free ORS; both the experimental and control solutions were rice-based and glucose-free [32]. In another study, a glucose-free peptilose-based ORS was found more advantageous and acceptable than the WHO-ORS for the treatment of mostly acute watery diarrhea among children [33].

It has been observed that certain amino acid-based pathways for sodium transport become the dominant pathway to compensate during times of stress [34]. These pathways can be utilized to rehydrate the intestinal epithelial cells and restore normal bowel function. Eight specific amino acids retained their absorptive capacity from the gut lumen following radiation and at the same time decreased paracellular permeability: lysine, aspartic acid, glycine, isoleucine, threonine, tyrosine, valine, and serine.

Enterade® is a proprietary blend of five amino acids (threonine, valine, serine, tyrosine, and tryptophan) [6, 7] used prophylactically as a treatment, Enterade modulates intestinal transmembrane proteins to promote intestinotrophic villus regrowth, increase sodium and water absorption, decrease chloride and bicarbonate secretion, and reduce intestinal paracellular permeability [7, 35]. The benefits of Enterade have been demonstrated by improvements in body weight maintenance and survival in mice with radiation enteritis and improved diarrhea outcomes in oncology patients suffering from toxic gut syndrome [6].

In a multi-center randomized study within a small subset of subjects who were able to maintain compliance with the study intervention, an exploratory analysis showed significantly less severe diarrhea with Enterade® among patients receiving high-dose chemotherapy [36]. A retrospective chart review of patients with neuroendocrine tumors shows the potential antidiarrheal activity of Enterade® Advanced Oncology Formula [35].

Entrinsic Bioscience (EBS) has developed a beverage (VS002) to restore water and electrolytes using amino acids to transport sodium and water without stimulating chloride secretion. Like glucose, EBS amino acids simultaneously also increase intestinal sodium flux through increased epithelial NHE3 (sodium-hydrogen antiporter 3). Unlike glucose, VS002 also decreases CFTR expression, which is an intestinal target protein that becomes heavily stimulated in response to infectious diarrhea [37]. As a consequence of increasing absorption and decreasing secretion, net intestinal absorption is superior to VS002 was used to treat diarrhea. Trioral® ORS (oral rehydration salts: WHO new formula for food poisoning, hangovers, diarrhea, electrolyte replacements) works in healthy and diseased (Cholera) rat intestine (*n*= 3 to 8 animals per trial) using single-pass intestinal perfusion (ex vivo) [unpublished data]. The diarrhea-diseased intestine exhibited reduced net absorption when compared with the healthy intestine (i.e., saline only). Net absorption in the diseased intestine was improved by the use of Trioral® ORS and even further improved by the use of VS002. The greater net absorption with VS002 relative to Trioral® can be explained at least in part by the additional chloride secretion activity of even very small amounts of glucose present in Trioral®, which has been demonstrated using chamber ^36^Cl and ^24^Na flux studies [30, 38], and an increase in NHE3 (sodium absorption) and decrease in cystic fibrosis transmembrane conductance regulator (CFTR) (chloride secretion) activity in intestinal tissues incubated in VS002 and measured using western blot analysis [37].

Studies with VS002 have shown that VS002 administration rapidly rehydrates to increase fluid and electrolyte absorption, tightens the mucosal barrier, thus decreasing local and systemic inflammation, and increases crypt count and villus height, leading to an increase in the surface area of absorption (unpublished data). There is no toxicity associated with VS002 administration and the results of studies in animals and humans suggest that VS002 can serve as a highly safe and effective supportive care in place of standard ORS [8, 39–41].

Trials in healthy humans demonstrated VS002 safety; and rehydration efficacy compared to a variety of commercial, glucose-containing beverages (sports drink, ORS) [42]. These studies included adults with diarrhea, healthy soldiers, young college students, and older adults (> 60 years old). Thus, administration of VS002 using eight amino acids to increase intestinal water and electrolyte absorption was associated with safe rehydration. VS002 was provided to > 250 adults treated for diarrhea in Indonesia and the Philippines. The consumption of VS002 was well tolerated by all subjects, with no adverse events [43, 44].

Traditional ORS contain sugars that stimulate intestinal sodium and water absorption through a variety of mechanisms. However, it has been under-appreciated that traditional ORS possess no anti-diarrheal functions and may exacerbate infectious diarrheal secretions (Yin et al., 2017). Even very small concentrations of glucose also produce a net secretion of chloride in the intestine. The mechanism for this observation is that glucose stimulates anoctamin 1 expression, which activates calcium-activated chloride channels [30].

By using this concept, the University of Florida (UF), developed a sugar-free, shelf-stable amino acid-based hydration medicinal food named “VS002A” that effectively rehydrates, and improves the barrier function of the bowel following infections targeting the gastrointestinal tract. EBS used a secretagogue model, which resulted in VS002A (aspartic acid, arginine, serine, proline, threonine, glycine, alanine, and tyrosine). VS002A has 5 amino acids (Aspartic acid, Glycine, Serine, Threonine, and Tyrosine) in common with VS002; 3 amino acids (Isoleucine, Lysine, and Valine) in VS002 are substituted by 3 other amino acids (Alanine, Arginine, and Proline) in VS002A, and the total mM concentration and gram weight (g/L) of VS002A are identical to VS002. All other ingredients in VS002A (e.g., electrolytes, flavors) are identical also to VS002. Therefore, it is hypothesized that the performance of VS002A will be substantially improved over VS002 and radically improved over WHO-ORS in the treatment of acute watery non-cholera diarrhea in infants and young children.

### Trial status

Recruitment of the VS002A trial began in June 2021 and is currently ongoing. It is expected to conclude in September 2022. The current protocol version 1.079 and is dated 22 May 2021.

### Limitations

This study has its limitations. As this is a single-center trial, among the male child with some dehydration, study findings may not be generalizable to other settings.

## Supplementary Information

**Additional file 1.**

## Data Availability

The datasets generated during the current study will be available from the corresponding author on reasonable request.
