# Supplementary material for: A double-blind clinical trial to compare the efficacy and safety of a multiple amino acid-based ORS with the standard WHO-ORS in the management of non-cholera acute watery diarrhea in infants and young children: “VS002A” trial protocol
Source: Trials. 2022 Aug 25;23:706. doi: 10.1186/s13063-022-06601-5 (PMC9403960; doi:10.1186/s13063-022-06601-5)
Supplement: Supplementary file 1 — Additional file 1. [file 13063_2022_6601_MOESM1_ESM.docx]

## Title: A double-blind clinical trial to compare the efficacy and safety of a multiple amino-acid based ORS with the standard WHO-ORS in the management of non-cholera acute watery diarrhea in infants and young children: “VS002A” trial protocol

## Safety data of Advanced amino acid-based ORS: VS002A

## The food additive amino acids may be safely used as nutrients added to foods as individual amino acids in the free, hydrated, or anhydrous form, or as the hydrochloride, sodium, or potassium salts:

## (1) L-Alanine; (2) L-Arginine; (3) L-Asparagine; (4) L-Aspartic acid; (5) L-Cysteine; (6) L-Cystine; (7) L-Glutamic acid; (8) L-Glutamine; (9) Aminoacetic acid (glycine); (10) L-Histidine; (11) L- Isoleucine; (12) L-Leucine; (13) L-Lysine; (14) DL-Methionine (not for infant foods); (15) L- Methionine; (16) L-Phenylalanine; (17) L-Proline; (18) L-Serine; (19) L-Threonine; (20) L-Tryptophan; (21) L-Tyrosine; (22) L-Valine. EBS amino acid formulas are fully compliant with FDA 21 CFR requirements.

## Both VS002 and VS002A are sugar free and contain amino acid blends with electrolytes. Entrinsic Bioscience, Inc., already manufactures a commercial 5 amino acid formula (enterade® AO) and 8 amino acid formula (enterade® DH) (Table 1). VS002A is a new formula not currently intended for commercial use in the U.S. or Bangladesh, but is being donated to icddr, b for clinical research purposes. Table 1 provides a comparison of the amino acid profiles for each. Other differences relate to the levels of electrolytes only.

| **Table 1. Comparison of EBS AA beverage formulas** | | | |
| --- | --- | --- | --- |
| *Amino Acid Information* | *enterade® AO* | *enterade® DH* | *VS002A* |
| Amino acids by weight | valine, aspartic acid, serine, threonine, tyrosine | valine, aspartic acid, serine, isoleucine, threonine, lysine,  glycine, tyrosine | aspartic acid, arginine, serine, proline, threonine, glycine,  alanine, tyrosine |
| Total Concentration (mM) | 37.2 | 57.2 | 57.2 |
| Total Gram Weight (g/L) | 4.5 | 6.8 | 6.8 |
| AO = Advanced Oncology; DH = Digestive Health | | | |

## Tables 2 and 3 reference the U.S. dietary mineral and amino acid intake guidelines for children ages 6 months to 3 years for comparison against what would be ingested when treating diarrhea in accordance with World Health Organization guidance under diarrhea treatment plan B. To address dosing of ingredients, Tables 2 and 3 show the maximum anticipated daily dose of each ingredient when consuming between 450 mL and 1800 mL of VS002A oral rehydration solution (ORS) relative to the U.S. Dietary Reference Intake (DRI) for each nutrient.

| **Table 2. Dietary Reference Intakes as Safety Criteria: Minerals** | | | | | |
| --- | --- | --- | --- | --- | --- |
| Nutrient | Sodium mg | Potassium mg | Chloride mg | Calcium mg | Magnesium mg |
| DRI1 | 110 –  800 | 400 –  2000 | 180 –  1500 | 200 –  700 | 30 – 80 |
| VS002A2 | 693 –  2772 | 351 –  1404 | 772 –  3087 | 22 – 88 | 5 – 18 |
| 1Dietary Reference Intakes (1997; 2005; 2011; 2019); children ages 6 months to 3 years  2Assumptions:   1. Range of child body weights from 6 kg to 12 kg 2. 75 mL per kg body weight per four-hour dosing schedule (IAW WHO Treatment Plan B) 3. Low-end range is for 6 kg over four hours (450 mL) and high-end range for 12 kg dosed for two iterations of 4 hours (1800 mL) 4. Table values calculated from EBS formula specification sheet and may differ slightly from bottle label values due to US FDA Nutrition Labeling Education Act rounding requirements   *Note: The same ranges of VS002A (above) also provide 1191 mg – 4765 mg of citrate (21CFR582.1751), 16 mg – 64 mg stevia leaf extract (21CFR170.36) (~5 mg/kg), and 810 mg – 3240 mg of flavors, all of which are Generally Recognized as Safe by the FDA as food additives; sodium and chloride values in excess of DRI are acceptable as deficit*  *therapy* | | | | | |

| **Table 3. Dietary Reference Intakes as Safety Criteria: Amino Acids and Total Protein** | | | | | | | | | |
| --- | --- | --- | --- | --- | --- | --- | --- | --- | --- |
| Nutrient | Aspartic Acid  mg | Threonine mg | Tyrosine mg | Glycine mg | Serine mg | Arginine mg | Alanine mg | Proline mg | Total Protein1  G |
| DRI1,3,4 | n/a3 | 380 – 440 | n/a3 | n/a3 | n/a3 | n/a3 | n/a3 | n/a3 | 9 – 13 |
| VS002A2 | 600 –  2390 | 430 –  1710 | 100 –  400 | 240 –  950 | 470 –  1890 | 530 –  2110 | 210 –  850 | 470 –  1870 | 3 – 12 |
| 1Dietary Reference Intakes for Macronutrients (2005), children ages 6 months to 3 years  2Assumptions:   1. Range of child body weights from 6 kg to 12 kg 2. 75 mL per kg body weight per four-hour dosing schedule (IAW WHO Treatment Plan B) 3. Low-end range is for 6 kg over four hours (450 mL) and high-end range for 12 kg dosed for two iterations of 4 hours (1800 mL) 4. Table values calculated from EBS formula specification sheet and may differ slightly from bottle label values due to US FDA Nutrition Labeling Education Act rounding requirements   3n/a indicates no DRI (dispensable amino acids)  *Note: threonine exceeds the DRI, but there are no adverse or toxic effects at the doses listed; total protein (and renal / osmotic solute load) are below the DRI* | | | | | | | | | |
